# Animal welfare, consumer welfare, and competition law: The Dutch debate on the Chicken of Tomorrow

**DOI:** 10.1093/af/vfx001

**Published:** 2018-04-11

**Authors:** Jacqueline M Bos, Henk van den Belt, Peter H Feindt

**Affiliations:** 1 Social Sciences Group, Centre for Communication, Philosophy, and Technology, Wageningen University, Wageningen, The Netherlands; 2 Strategic Communication Group, Wageningen University, Wageningen, The Netherlands; 3 Humboldt-Universität zu Berlin, Albrecht Daniel Thaer-Institut für Agrar-und Gartenbauwissenschaften, Berlin, Germany

**Keywords:** animal welfare, chicken meat production, Dutch Consumer and Market Authority, horizontal agreements, sustainability initiatives

**Implications**

- The business sector is often expected to innovate in promoting sustainable animal-sourced products. However, the Dutch Consumer and Market Authority ruled that a voluntary arrangement for a more ethical and sustainable chicken meat concept, the Chicken of Tomorrow, violated national and European Union competition law.
- The ensuing debate on the Chicken of Tomorrow meat concept revealed that consumer acceptance of such sustainability agreements would depend on conditional self-regulation which is based on the explicit articulation of and alignment with public interests.

## Introduction

Business initiatives to create markets for ethical products such as animal-friendly meat products or child labor-free clothing may be restrained by competition law if they involve agreements that reduce consumer choice. In the Netherlands, supermarkets, poultry farmers, and chicken meat processors agreed in 2015 on a chicken meat concept with an enhanced sustainability profile: the ‘Chicken of Tomorrow’. It established private animal welfare standards above the legally required minimum, such as the use of a slower growing breed, more space in the poultry house, natural day and night rhythm, and provision of distraction materials. Chicken of Tomorrow also addresses public health and environmental concerns through less use of antibiotics, “responsible soy” in feed, reduced emissions of ammonia and particulates, and closed mineral cycles. The goal of this agreement was that by 2020 supermarkets would sell fresh chicken meat exclusively from animals that have been produced under these improved conditions. Accordingly, supermarkets would offer higher purchasing prices to meat processors and poultry farmers positioned further up the supply chain. However, the Dutch Consumer and Market Authority (**ACM**) ruled that these arrangements violated national and European Union (EU) competition rules (ACM, 2015). According to ACM, the benefits in terms of animal welfare and sustainability did not outweigh the disadvantages for consumers from limited choice and a higher product prices. The decision highlighted a conflict between consumer protection and animal welfare goals.

Public opinion and the Dutch government expect the private sector to innovate in promoting sustainable animal-sourced products (Ministry of Economic Affairs, 2013). This works well when efficiency savings can be made that benefit companies and consumers. However, further progress requires the agro-food sector to also internalize environmental and social externalities, which would lead to higher product prices and the removal of the least sustainable products from the market. Supermarket chains face the dilemma of either advancing alone and risking the loss of price-oriented customers or collaborating with their competitors in potential breach of competition law for conspiring to raise prices or to limit consumer choice. The strict approach of the ACM caused much consternation among participants in the Chicken of Tomorrow initiative and other civil society organizations, environmental and animal welfare NGOs.

**Figure:**
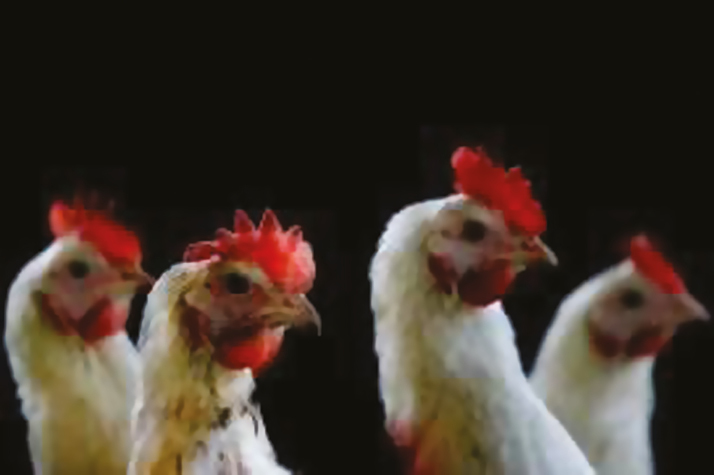
(a) Source: Distrifood

**Figure:**
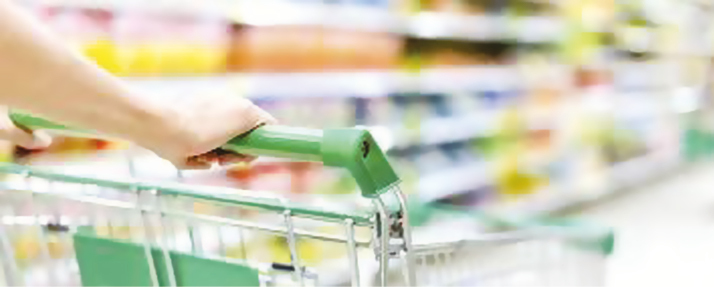
(b) Source: Plukon

In response, the Minister of Economic Affairs issued an “adjusted policy rule” to “clarify and explicitly guide the competition authority ACM in assessing sustainability initiatives” (Ministry of Economic Affairs, 2015, 2016). Notably, the Dutch competition law protects merely “consumers’ interest” rather than a broader concept of “public interest.” Meanwhile, how the consumers’ interest should be interpreted is fiercely debated. Besides traditional considerations of choice, price, and innovation, broader definitions include sustainability concerns. Legal debates are divided between economists, who tend to focus on price, and other social scientists, philosophers, and lawyers who espouse broader interpretations. This article analyses the Dutch debate on legally acceptable ways to incorporate animal welfare goals in governance and responsible business conduct and the mandate of the competition authority and assesses the ethical and political significance of the (provisional) outcomes.

## The Perspective of Competition Law

The law on cartels and horizontal competition is enshrined in Article 101 of the Treaty on the Functioning of the European Union (**TFEU**) and in Article 6 of the Dutch Competition Act: the latter applies to agreements that only comprise the Netherlands, Article 101 TFEU to those that “affect trade between Member States.” Both provisions prohibit any agreement between companies that restricts, prevents, or distorts competition, unless it “contributes to improving the production or distribution of goods or to promoting technical or economic progress, while allowing consumers a fair share of the resulting benefit” (art. 101, 3 TFEU). To escape annulment, agreements therefore need to be able to demonstrate either that they do not restrict competition or that they are indispensable to achieve the desirable aims while providing fair benefits to consumers. Failure to provide such evidence can result in fines and even in prison sentences. Competition-inhibiting agreements that introduce minimum environmental or nutritional standards could still be allowed if an overriding consumer benefit can be demonstrated (Food Ethics Council, 2011).

In the Netherlands, a vision document by the ACM (2014) introduced a “broad welfare concept” that, while based on consumer preferences, recognized a possible inclusion of environmentally friendly and animal-friendly modes of production. The ACM also conceded that coordination problems might impede the introduction of more sustainable products: “the existence of a first-mover disadvantage could be an argument […] to consider a market-wide arrangement justified” (ACM, 2014, § 3.5.3).

However, the broader future benefits still have to be discounted, according to guidelines by the European Commission (2004, § 88). Similarly, while benefits might accrue to consumers not directly affected, the latter “cannot be worse off as a result of the arrangement” (ACM, 2014, § 3.5.2; cf. European Commission, 2004, § 85). Furthermore, the European Commission stated “that it is indeed possible to take future benefits for consumers into account, but that paragraph 3 of Article 101 of the EU Treaty requires that the benefits accrue to the actual users of the relevant goods and services (and that it is not acceptable, within this framework, to include the benefits for society as a whole)” (Ministry of Economic Affairs, 2014, § 4).

The most important limitation of the ACM’s consumer welfare approach is that sustainability gains are not automatically considered as welfare gains unless this is perceived by consumers as “value creation,” preferably to be proven by a revealed willingness to pay (**WTP**):

Many consumers are willing to pay more for sustainable products because they value the realization of the ideas behind such products. When determining the benefits, qualitative improvements that create value through the introduction of new or improved products may thus be taken into consideration. When arrangements simultaneously lead to a reduction of supply—for example, by taking animal-unfriendly products off the market—it is necessary that it can be demonstrated that the (new) supply really is an improvement in terms of quality, or that it is at least perceived as such by consumers. The latter may be demonstrated by the willingness of a substantial share of consumers to possibly pay more for these products. (ACM, 2014, § 3.5.1)

Notably, the ACM does not require proof that “animal-friendly” products actually improve the welfare of production animals but would be content with perceived improvements. In search for standards for demonstrable evidence, the ACM then opts for consumer WTP. In the ACM’s view, “taking animal-unfriendly products off the market” cannot be allowed as a matter of course, but only under stringent conditions. Following an exchange between ACM and the European Commission over the exemption of cartel prohibition for the Chicken of Tomorrow and similar initiatives, the ACM guidelines require as a precondition for such an exemption the proof of perceived benefits to some affected consumers and the absence of disadvantages for all affected consumers. The perceived benefits have to be quantified in monetary terms. In the past, similar arrangements have been banned because they directly affected the retail price at supermarkets.

**Figure:**
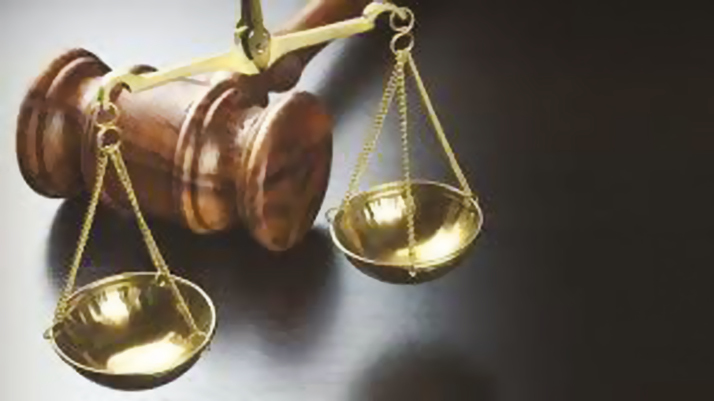
(c) Source: Boerenbusiness Description (a), (b) and (c): Animal welfare, consumer welfare and competition law.

For example, in the “milk-dime” case in 2001, several supermarket chains in the Netherlands requested permission to charge consumers an additional 10 cents per liter of milk to compensate the increased purchasing costs after an outbreak of foot-and-mouth disease. The Dutch competition authority refused permission. In 2015, the ACM found the Chicken of Tomorrow agreement to restrict competition, as it encompassed all supermarket chains in the Netherlands which together cover 95% of the country’s sales of fresh chicken meat. Therefore, the agreement to remove regular chicken meat from their shelves was seen as limiting consumers’ choice. But the ACM also had to assess whether the exemption criteria set out in article 101, 3 TFEU and in article 6, 3 Dutch Competition Act that could justify the agreement were met. The ACM (2015) summarized these exemption criteria in four steps:

1. The anticompetitive arrangement must contribute to the improvement of production or distribution, or to the promotion of technical or economic progress;
2. Consumers have to receive a “fair share” of these benefits;
3. The anticompetitive arrangement must be necessary and proportional to the attainment of the efficiencies that are realized by the arrangement; and
4. Sufficient residual competition must continue to exist in the market.

In essence, animal welfare initiatives in the form of horizontal agreements among retailers that categorize specific products as low in animal welfare and remove them from the market have to demonstrate that this reduction in consumer choice creates a measurable benefit to consumers.

## Standards of Evidence: Consumer WTP

To determine whether the Chicken of Tomorrow arrangement was in the interest of consumers, the ACM asked its Bureau of the Chief Economist to conduct a cost–benefit analysis that also included a survey study into the consumer WTP (Bureau ACM, 2014). To determine the value attached by consumers to certain (credence) attributes of the Chicken of Tomorrow concept, the survey study asked 1,603 participants to make a series of choices by indicating their preferences within a controlled set of products from broiler chicken raised under different circumstances. The analysis of these choices revealed the implicit valuation of individual elements making up the meat product. On the basis of this “choice-based conjoint method” (Lagervist and Hess, 2010), the researchers estimated the WTP for various underlying attributes (e.g., broiler life span to slaughter, occupancy rate in the poultry barn, type of animal feed, availability of a covered outdoor area, and type of anesthesia at the slaughterhouse). This allowed to calculate the WTP for the different conditions (or standards) in various broiler husbandry systems (Table 1): regular (meeting basic requirements), Chicken of Tomorrow (new Dutch standard), one star of the Better Life Hallmark of the Dutch Society for the Protection of Animals (free range), and organic (Bureau ACM, 2014).

**Figure:**
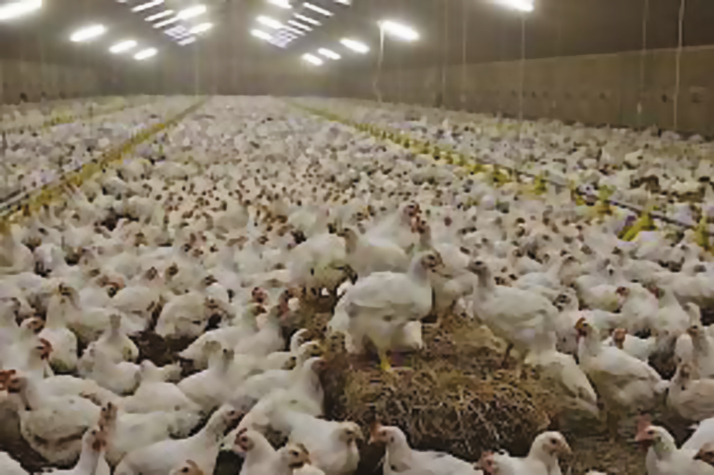
(e) Source: Pluimveeweb

**Figure:**
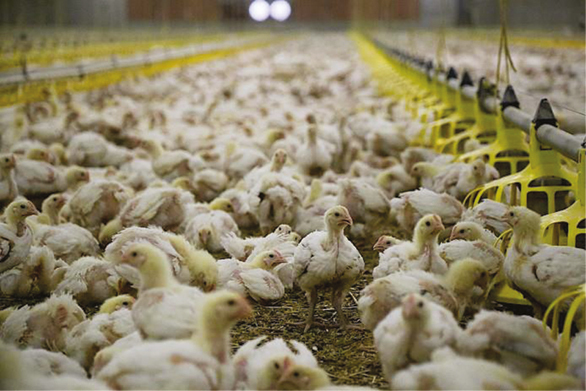
(f) Source: Pluimveeweb

**Table 1.** Comparison of standards in various broiler husbandry systems: regular, Chicken of Tomorrow, and one star Better Life Hallmark.

Standards Broiler breed	Regular *Ross 308*	Chicken of Tomorrow *Hubbard JA 987*	One star Better Life Hallmark *Hubbard JA 757*
Occupacy rate per square meter	21 broilers 42 kg	19 broilers 38 kg	12 broilers 25 kg
Covered outdoor areas	No	No	Yes
Growth rate per day	66 g	50 g	42 g
Life span before slaughter	37–42 d	49 d	56 d

Sources: Dierenbescherming, LEI Wageningen University, Albert Heijn, Plukon.

The analysis found that the additional WTP for the Chicken of Tomorrow compared with regular chicken meat was 68 eurocent/kg. In contrast, the extra WTP for the one star Better Life Hallmark (free range) compared with regular chicken meat was 11.99 euro/kg. The WTP survey study concluded that consumers (i.e., research participants to the survey) were not particularly susceptible to the animal welfare criteria in the Chicken of Tomorrow concept (increased life span before slaughter, lower occupancy rate in poultry house, etc.), but attached higher value to the availability of covered outdoor areas for broiler chicken raised under the one star Better Life Hallmark. The Chicken of Tomorrow concept also earned a modest increase in WTP of 14 eurocent/kg for environmental aspects such as animal feed according to the Round Table of Responsible Soy. According to the ACMs’ Bureau of the Chief Economist, the total extra WTP (68 + 14 = 82 eurocent) could not offset the calculated additional price of 1.46 euro/kg asked from consumers in the supermarket (Bureau ACM, 2014).

The ACM’s Economist’s claim was contested by legal experts. Jochemsen-Vernooij and Van der Heul (2015) pointed out that the Chicken of Tomorrow agreement was about supermarkets adjusting their purchasing conditions towards meat processors and poultry farmers. This implied that the calculated higher price should be regarded as the valuation of the purchasing conditions instead of what the ACM considered as the retail price in the supermarket. The authors also criticized the ACM for disregarding the possible applicability of the “doctrine of inherent limitations.” Intense academic debate focused on the proper normative foundations of European competition law, especially since the European Commission established consumer welfare as the overriding criterion in legal assessments of exemptions to the cartel prohibition (Dubbink and Van der Putten, 2008; Kingston, 2009, 2010; Baarsma, 2011; Gerbrandy, 2013; Claassen and Gerbrandy, 2016).

One line of critique follows the American philosopher Mark Sagoff who broadly explored public policy and societal decision-making within ethically sensitive areas (Sagoff, 1989). Sagoff (1989, p. 10) argues that the use of traditional cost–benefit economics to assess values in the environment, health care, and other public domains constitutes a categorical mistake (confusion of the kinds of value that are included in deliberations). Animal ethicist Jess Harfeld (2010, 2013) emphasized that approaching the animal merely in the system of the market inevitably implies an evaluative mistake, i.e., a categorical mistake about the kinds of value that are included in deliberations.

Similarly, it can be argued that consumer welfare as the leading standard is inappropriately narrow because it does not allow for the mandated integration of environmental goals in all policy areas (Townley, 2007; Gerbrandy, 2013; Daskalova, 2015). If the value of a broiler chicken can be determined only in terms of monetary market value, participants are unable to talk meaningfully about “the intrinsic value” of farm animals (Regan, 1985), i.e., that animals are ends-in-themselves (Harfeld, 2013, p. 265). WTP analysis aims to translate into a one-dimensional monetary valuation the complexity of aesthetical, societal, and ethical values that are at play when people evaluate options and make decisions within the public domain (Harfeld, 2010, p. 137). This academic criticism, however, had little effect in challenging the dominance of the consumer welfare standard in the decision practice of national competition authorities.

**Figure:**
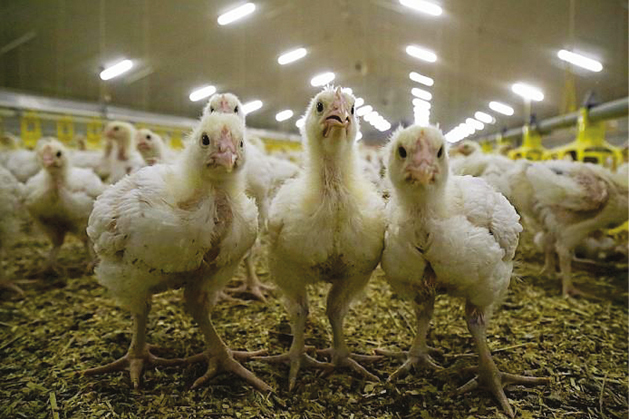
(g) Source: Pluimveeweb

With respect to the Chicken of Tomorrow, the ACM’s general conclusion was that the initiative violated the standards of competition law. First, it brought no net benefits to consumers and therefore failed to pass the main test. Second, from the unexpected high extra WTP for the one star Better Life Standard, the ACM concluded that retailers and other businesses had actually failed to grasp the economic opportunities at hand. Third, given the high WTP for Better Life standard products, each individual supermarket chain should be able to profitably differentiate itself from the competitors in the market through distinct vertical agreements in their respective chicken meat supply chain. Therefore, the ACM did not find a need for a horizontal market-wide agreement, for instance to overcome a “first-mover disadvantage” (ACM, 2015), and ruled the Chicken of Tomorrow agreement as contrary to competition law.

## Competition Law: Weighing Public Interests

The approach of the competition authority ACM implies that companies and civil society organizations who want to use horizontal agreements to improve sustainability or animal welfare may find their plans unfeasible. As the Dutch government aims to provide more room to companies that jointly make sustainability agreements, the Minister of Economic Affairs deemed the policy on competition and sustainability (which included animal welfare) and the guidance on the interpretation of the Competition Act in need of adjustment (Ministry of Economic Affairs, 2015, 2016).

**Figure:**
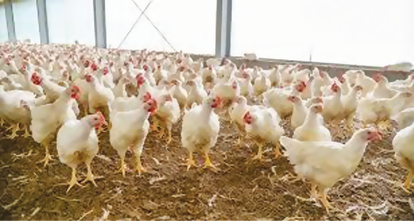
(h) Source: De Heus Description for (h): The availability of covered outdoor areas for broiler chicken raised under one star Better Life Hallmark.

**Figure:**
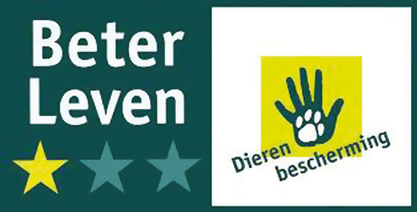
(i) Source: Dierenbescherming

The draft version of the Ministry’s new policy rule has been submitted for public consultation in 2015 and 2017 (Ministry of Economic Affairs, 2015, 2017). One of the challenges that the Dutch government faces in the reduction of barriers defined by competition law with regard to public interest- oriented collaborative initiatives is the independence of ACM as a public body. The independence of the ACM as a public body implies that there is no formal alignment between government officials and ministers pushing particular environmental and social objectives and the competition watchdog. The current policy therefore aims to “clarify and explicitly guide the ACM in assessing sustainability initiatives.”

The draft new guidance was discussed by various civil society organizations and by the Social Economic Council (**SER**), an advisory board to the government that includes representatives of employers and workers associations along independent experts. The SER, who consulted the Nature & Environment Association and the Consumer Association, advised to extend the scope of the existing policy (SER, 2016, 2017) and to include the “doctrine of inherent constraints” to harmonize voluntary sustainability agreements with competition law. This doctrine states that if a restriction of competition is necessary and proportional to its public interest purpose, the prohibition normally required by competition law should not apply. The doctrine derives from statements by the European Court of Justice which has repeatedly found a restriction of competition acceptable if it served a legitimate public interest.

The ACM had stated that improving the well-being of broiler chicken was the task of policymakers who should use regulation to pursue societal goals (Weissink, 2015). In contrast, the SER called for a clarification of the relevant “public interest” rather than adding more regulation: the route of “conditioned self-regulation,” i.e., self-regulation under specified conditions (SER, 2016, 2017). It thereby called for a move to a controlled release of regulative systems, i.e., “the government deliberately allowing or even stimulating forms of self-regulation, while simultaneously subjecting these to certain conditions” (SER, 2016).

The Dutch Bar Association NOvA emphasized the limited mandate of the ACM in its response to the draft policy rule. It also endorsed the concept of conditioned self-regulation (Nederlandse Orde van Advocaten, 2016). The NOvA argued that a one-sided focus on the exemption criteria to the cartel prohibition would give an excessively large role to the competition authority and would lead to subsuming public interests under the economic criterion of consumer welfare, however “broad” that concept might be taken. Therefore, to avoid that the ACM determines what is basically an ethical standard or a public interest, policymakers in government and parliament would have the task to clearly explain and articulate the relevant public interest. The SER adopted a similar perspective by calling for an explicit description of public interests in the governments’ official documents, such as the government policy accord, policy papers, or responses to questions from members of Parliament. The SER embraces the view that the new policy rule may benefit from a pragmatic compliance with already existing international mandatory agreements such as OECD guidelines for multinational companies. These agreements represent already established standards of public interest.

The SER also sees a need for the participation of stakeholders and civil society organizations in the establishment of relevant ethical standards through deliberative processes (SER, 2016, 2017). The competition authority can only assess the legitimate purpose of a sustainability or animal welfare initiative if such deliberations are nurtured ex ante. This would put the assessment of the need for anticompetitive arrangements and their proportionality in terms of the desired purpose on a more inclusive and deliberate basis.

## Discussion

What lessons can be drawn from the experience of the Chicken of Tomorrow? Based on a 2015 “consumer willingness to pay” survey, the ACM concluded that the Chicken-of-Tomorrow benefits in terms of animal welfare were limited. As such, it concluded that the perceived benefits did not justify the disadvantages for consumers in terms of limited choice and higher retail price. Nevertheless, in July 2017, the Dutch supermarket chain Albert Hein reported a market share of 76% of their New AH Standard chicken (their home brand following the Chicken of Tomorrow concept), compared with 3% organic, 20% free range (one star of the Better Life Hallmark), and 1% miscellaneous (Graumans, 2017). By July 2017, all the supermarket chains in the Netherlands had developed their own home brand in accordance with the Chicken of Tomorrow concept (Poultryworld, 2017; Van Ammelrooy, 2017). In the absence of a horizontal agreement, this development is in line with the judgment and recommendations of the ACM in 2015.

**Figure:**
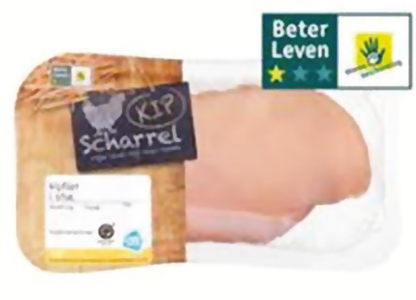
(j) Source: Albert Heijn Description for (i) and (j): One star of the Better Life Hallmark of the Dutch Society for the Protection of Animals on free range chicken meat.

In spite of the issues articulated in this article, the retail sector embraced the Chicken of Tomorrow as an effective resolution of the existing conceptual and politico-economic stalemate between competition and sustainability. The ensuing articulation of a shared concept helped to standardize and communicate improved animal welfare practice even in the absence of a formal agreement. As an effect, the participating supermarket chains now escape the previous aggressive criticism from the Dutch animal welfare organization Wakker Dier whose vigilant naming and shaming strategy had targeted retailers who promote and sell cheap (regular) fresh chicken meat from faster growing broilers (Wakker Dier, 2017). Still, whether the goal of animal welfare has been fully achieved through the embrace of the Chicken of Tomorrow remains open for debate.

Many stakeholders in the poultry industry and retail claim that consumers will not easily move away from more animal-friendly chicken meat products since their production entails a higher consumer price. Here we might return to Sagoff’s (1989, p. 10) critique of cost–benefit economics as a categorical mistake when applied to the assessment of functional values in public domains and the way in which these inform consumer choice. Consumers and citizens have different roles (cf. Harfeld, 2010, 2013), and the choices a citizen makes in the supermarket may not be in line with his or her opinions as a citizen.

Firstly, consumers—or consuming citizens—are embedded in a system of markets and consumption practices that is often taken for granted. In the official Dutch discussion about animal-friendly chicken production, national and global agricultural markets remain mostly unquestioned. According to Harfeld (2013, p. 265), this complicates the conceptualization of animals in agricultural market systems. An ethical approach that assigns intrinsic value to animals is therefore not self- evidently part of producers’ views on consumer choice, while there could be room for the creation of new markets on this basis.

Second, as Harfeld explains, there are two different roles through which the inhabitants of any given society are motivated and act: the consumer and the citizen. Human beings can act in ways that are purely individualistic based on self-interest. This is what Sagoff calls the consumer side of people. In contrast, as citizens, people are concerned not only with their own wants and interests but rather “with the public interest” (Sagoff, 1989). In their role as citizens people are enabled and expected to act out of concern for society as a whole (Harfeld, 2010, p. 137). In a similar way, the difference between the consumer and the citizen could be understood as the difference between wants and values. At the level of wants, the Chicken of Tomorrow concept has a market value that can be expressed in economic language. Wants are readily accessible through the concept of WTP. However, this is not equivalent to ascribing intrinsic value to a person, an animal, an object, and an experience such as love, salvation, cultural legacies, and natural wonders (Harfeld, 2010, pp. 138–139).

Third, the participants to the Chicken of Tomorrow initiative were all representatives from the agro-food sector, namely poultry farmers, meat processors, and supermarkets. The initiative did not include a broader range of civil society organizations, for instance environmental protection organizations, the Dutch organization for the protection of animals, or consumer associations. Inclusion of these organizations in deliberations would help to fully articulate the relevant public interests and weigh them in a more integrative manner: affordable prices, consumer choice, product innovation, public health, and criteria for animal welfare and environmental protection.

Fourth, an ethical evaluation of the circumstances under which broiler chicken are raised cannot only be directed at individuals and their actions and attitudes. The issue of animal-friendly meat production is also a matter of addressing the situations and problems at the level of communities, social structures, and policies, which is not a question of individual reflection but rather of political philosophy (Harfeld, 2010, p. 158). More particularly, the responsibilities and the systemic interplay of the actions of a range of persons and organizations—farmers, meat processors, retailers, and consumers—need to be considered, as all of them make critical choices in food production, sale, and purchase that affect the welfare of the production animals.

## Conclusion

The decision of the Dutch competition authority to disallow horizontal agreements that would remove chicken products with relatively low but still legal levels of animal welfare is a case that exemplifies structurally embedded difficulties 1) in creating an institutional framework that enables a systemic approach to the improvement of animal welfare above legal minimum standards and 2) in establishing rules of evidence that acknowledge the ethical standards established by the animal welfare approach. Instead, in this case, the institutional framework prioritized consumer choice over animal welfare above the legal minimum requirements.

The establishment of consumer WTP as the decisive concept for creating evidence subsumed animal welfare under consumer value, thereby constructing animal welfare as an argument in a consumer welfare function. This understanding of animal welfare remains within an anthropocentric horizon and neglects ethical arguments for animal welfare that are grounded in the intrinsic value and the experience of the affected animals.

The processes of mass poultry production are increasingly scrutinized by the broader public. But while the Chicken of Tomorrow agreement was in line with an expressed societal consensus on improved animal welfare and consumers were still free to abstain from the purchase, animal welfare should not be used as a pretense to overcharge consumers. At the same time, the controversy over competition law has also backgrounded the lack of inclusion of civil society organizations. These organizations could be more helpful in the establishment and legitimation of relevant ethical standards. This would constitute an important step towards a more inclusive and transparent type of self-regulation that responds to societal demands for more animal welfare that are not easily translated into higher legal minimum standards or consumer WTP. It would also create new spaces for the actualization of the active citizen role in addition to the well-established roles as consumer in the supermarket and as subjects at the receiving end of governmental regulation.

## About the Authors

**Jacqueline M. Bos** is PhD candidate at Wageningen University, The Netherlands. Her PhD research focuses on the implementation of animal ethics through market creation for sustainable animal-sourced products.

**Dr Henk van den Belt** is assistant professor at Wageningen University, The Netherlands. He is especially interested in the ethics and philosophy of the modern life sciences.

**Prof. Dr Peter H. Feindt** was Professor of Strategic Communication at Wageningen University. Since October 2017, he is professor of Agricultural and Food Policy at Humboldt University at Berlin.
